# A machine learning based scheme for enhancing the detection of position falsification attacks in vehicular ad hoc networks

**DOI:** 10.1038/s41598-026-39867-9

**Published:** 2026-03-11

**Authors:** Eslam Abdelkreem, Sherif Hussein, Ashraf Tammam

**Affiliations:** 1 Faculty of Engineering, Computer Engineering Department, Arab Academy for Science Technology and Maritime Transport, Cairo, Egypt; 2 Computers and AI Department, Military Technical College, Cairo, Egypt

**Keywords:** VANET, Misbehavior detection, Position falsification, MDS, Machine learning, RSSI, Computer science, Information technology

## Abstract

Vehicular Ad Hoc Networks (VANETs) are wireless networks established between vehicles and their surrounding infrastructure, enabling the exchange of information. Consequently, many applications that can enhance passengers’ safety and traffic flow are built upon this information. However, malicious nodes can manipulate the exchanged data to attack other nodes and disrupt the network’s normal behavior. For example, if an attacker broadcasts a falsified location for a vehicle, the functionality of applications that rely on accurate location sharing will be compromised, potentially leading to deadly accidents. Although numerous Misbehavior Detection Schemes (MDSs) have been proposed to detect position falsification attacks, their effectiveness remains limited for certain attack types, raising concerns given the safety-critical nature of VANET applications. This paper proposes a machine learning-based method for detecting position falsification attacks. The proposed approach evaluates four machine-learning algorithms using three feature vectors (FV1, FV2, and FV3) composed of selected and derived features extracted from Basic Safety Messages (BSMs), in addition to a novel confidence-based Received Signal Strength Indicator feature, termed RSSIConf. The RSSIConf feature assesses the reliability of a sender’s claimed position by comparing the measured RSSI with confidence intervals corresponding to the claimed sender–receiver distance. Experimental results show that the Random Forest classifier trained with FV2 features achieves the best overall performance, outperforming existing approaches with improvements ranging from 0.76% to 13.26% in accuracy and from 0.74% to 12.71% in F1-score across different position spoofing attack types. These improvements enhance the reliability of misbehavior detection and contribute to safer and more trustworthy VANET communications.

## Introduction

Vehicular Ad Hoc Network (VANET) is a vital technology in implementing Intelligent Transportation Systems (ITS) that can help solve traffic and safety-related challenges. VANET creates a wireless network between vehicles and the surrounding objects that have communication capabilities^1,2^. Traditionally, VANETs relied on Dedicated Short-Range Communications (DSRC), which operates in the frequency range between 5.85 and 5.925 GHz that is segmented into seven channels, each of 10 MHz, and the remaining 5 MHz is for possible future needs^3^. Under DSRC, a message can propagate roughly 300 m to 1000 m and is either an Event-based alert or a Basic Safety Message (BSM)^4^. Event-based alerts are sent out when criteria are met, like lane changes and collisions^5^. In contrast, BSMs are broadcast every 100 ms to share vehicle-related information, like location coordinates, velocity, acceleration, and heading^6^. Recent advancements in vehicular communication technologies have introduced Cellular-Vehicle-to-anything (C-V2X) as a promising alternative to DSRC, offering enhanced reliability, range, and scalability for future ITS deployments^7^.

A wide range of applications can be developed using VANETs. These applications can be comfort-oriented or safety-oriented. Comfort-oriented applications aim to enhance passengers’ ease by offering entertainment features such as weather reports and the locations of nearby facilities^8^. In contrast, safety-oriented applications strengthen the protection of all road network users^9^. According to a road safety report created by the World Health Organization (WHO), more young people and children die because of traffic accidents than any other cause of death, including global diseases^10^. This number can be reduced using safety applications that utilize information shared through VANET communications to take the appropriate actions^11^. Such applications include lane change warnings, collision avoidance in intersections, and public safety applications^12^.

VANET is vulnerable to several security risks because of its characteristics, including high mobility, dynamic network topology, and open-access environment^13,14^. These security risks can be categorized into availability, data manipulation, confidentiality, and authentication attacks^15,16^. Such attacks can lead to incorrect decisions and severe accidents^17,18^. One of these attacks is position falsification, which can be carried out by broadcasting BSMs with falsified location information. This attack is considered one of the critical message tampering attacks as it can result in a disruption of road management and safety applications that rely on accurate position data of surrounding objects^19,20^. In addition, it can impair the performance of packet forwarding within the network^21^.

Thus, implementing systems that can detect and classify misbehaving nodes that launch this attack is essential^22^. These systems are known as Misbehavior Detection Schemes (MDSs). Although many prior studies have developed MDSs for location spoofing attacks, the detection efficiency of these models has been restricted when dealing with the different variants of that attack. For example, according to the survey, the highest reported performance for some position falsification attack types does not exceed 86.63% accuracy and 91.6% F1-score. Given the safety-critical nature of VANET applications, these performance levels are insufficient, indicating a clear need for more robust and accurate detection mechanisms.

Accordingly, the main objective of this research is to enhance the security of vehicular ad-hoc networks by proposing a machine learning (ML)–based misbehavior detection scheme capable of accurately identifying position falsification attacks. The key contributions presented in this paper are as follows:

- A new confidence-based Received Signal Strength Indicator (RSSI) feature, called RSSIConf, was proposed to capture inconsistencies between the claimed sender–receiver distance and the observed signal strength, thereby enhancing the detectability of position falsification attacks.
- Multiple ML-based detectors were designed and evaluated using different feature vectors and learning algorithms, and the most effective configuration for position falsification detection was identified through comprehensive performance analysis and comparison with existing approaches.
- An ablation study was conducted to quantitatively assess the contribution of the RSSIConf feature by comparing the proposed model against a modified version that excluded this feature under identical evaluation conditions.

The structure of the paper is as follows: “Related work” provides a survey of techniques for detecting position falsification in VANET, and “Proposed model” details the different implementation phases of the proposed model, including the introduction of a new feature, creation of different feature vectors, and training and testing of the models. “Evaluation and deployment” evaluates the proposed model and compares its efficiency to other models in the literature. The last section, “Conclusion and future work”, concludes the results and discusses possible future work.

## Related work

MDSs can be developed and implemented using traditional and ML techniques. Conventional detection methods use statistical and rule-based approaches that depend on pre-established rules and thresholds. In contrast to conventional techniques, ML-based methods can minimize human intervention, automate threshold selection, adapt to dynamic environments, learn from data, and identify unfamiliar attack patterns without being restricted by preset thresholds^23^.

The development and evaluation of MDSs in VANETs face several challenges. One of these challenges is acquiring a suitable log of messages exchanged between vehicles that can be used for implementing and testing the scheme. Real-world field studies can produce such messages; however, they are expensive and require specific tools^24^. As a result, several researchers utilize simulation techniques to generate these messages and build frameworks that ease the simulation process and enhance scalability^25–27^.

Furthermore, public datasets that simulate specific attacks have been developed and made available to researchers. For instance, the Vehicular Reference Misbehaviour Dataset (VeReMi) and the Burwood SUMO Traffic (BuST) are publicly available datasets that simulate multiple position falsification attacks in different scenarios^24,28^. The critical distinction between them is that the second is based on a traffic model that simulates a city in Australia with left-hand traffic restrictions. In^29^, the authors extended the VeReMi dataset to include additional attacks and introduced sensor errors.

The simulated attacks in VeReMi are Constant Position (CP), Constant Offset Position (COP), Random Position (RP), Random Offset Position (ROP), and Eventual Stop (ES). CP attackers send a fixed position during the simulation, whereas COP attackers add a constant distance to the accurate position. In RP, attackers send random positions for each message, while in ROP, they add random offsets to the actual positions before transmission. Finally, in the ES attack, attackers send a fixed position to simulate a sudden stop, then resume regular behavior.

VeReMi is commonly utilized in current research focusing on position-related attacks because it is publicly available, facilitates benchmarking of models, and provides multiple types of position falsification attacks. Moreover, the data generated by it were thoroughly validated to ensure the simulation’s realism^30^. Thus, the focus of this survey is on models that have utilized the VeReMi dataset, enabling an objective benchmarking process to identify the most effective approaches without bias. Furthermore, the proposed model is implemented using the same dataset, ensuring an unbiased evaluation against the benchmarked models.

### Traditional techniques

Ruj et al.^31^ proposed a misbehavior detection approach that focuses on verifying the consistency and plausibility of reported information. The scheme incorporates plausibility checks based on the physical constraints of vehicle movement and logical consistency rules. One key aspect of their method is the validation of the spatial relationship between the event location, the sender’s location, and the receiver’s location, ensuring the correct ordering and feasibility of the reported positions. The authors argue that in highly dynamic environments like VANETs, it is more practical and efficient to verify the correctness of the data than to determine whether a node is malicious.

In^32^, Abu-Elkheir et al. proposed a position verification scheme that relies on collaborative exchange of one-hop neighbor information to assess the plausibility of a vehicle’s announced location. By aggregating and analyzing two-hop neighbor data, the system constructs a logical view of neighborhood connectivity and traffic flow. A vehicle’s reported position is then evaluated against a plausibility area derived from this neighborhood context. If the claimed position falls outside the expected area, it may be flagged as false.

In^33^, the authors introduced a position verification method that combines several traditional plausibility checks, such as acceptance range, neighbor-table consistency, and velocity consistency, using a Subjective Logic framework. This approach moves beyond fixed decision rules by accounting for uncertainty in the input data, allowing it to make more flexible and reliable judgments about a vehicle’s claimed position. Instead of relying solely on strict thresholds, it considers different confidence levels, making it more adaptable to the variability found in real-world VANET environments. The method was tested using the VEINS simulator under various traffic conditions and attacker scenarios.

Kamel et al.^34^ presented an approach for misbehavior identification called CaTch, in which the uncertainty factor was integrated with several plausibility checks. The utilized checks include range plausibility, position plausibility, velocity plausibility, location consistency, velocity consistency, position-to-velocity consistency, and direction consistency.

In^30^, three physical-layer plausibility checks were presented utilizing the RSSI of BSMs. In the first check, the transmitting vehicle is instantly categorized as malicious once a BSM is outside the confidence interval of the RSSI and distance distribution. On the other hand, in the second check, the vehicle is categorized as misbehaving if most BSMs are outside that confidence interval. Finally, in the third check, each car receives a weighted score that is updated for each new BSM the vehicle gets.

In^35^, Pattanayak et al. used acceptance range, mobility grade, and maximum density thresholds to verify the vehicle’s claimed position. For instance, the acceptance range threshold assigns a maximum threshold value to determine the communication ranges of nodes inside the network. Similarly, the mobility grade threshold utilizes speed and location data to calculate acceleration and estimate node positions, identifying potentially malicious nodes that fall outside the specified distance threshold.

### ML-based techniques

In ML-based models, binary or multiclass classification strategies are possible^36^. Binary classifiers differentiate between two classes, one representing attackers and the other representing legitimate messages. On the other hand, multiclass classifiers categorize messages into multiple classes to detect the attack and identify its specific type. Based on the conducted survey, most schemes were developed for one of two modes:

- Binary Categorization for a Single Type (BCS): In the BCS mode, a separate model was implemented for each type of different position spoofing attack types.
- Binary Categorization for Many Types (BCM): In that case, the classifier implemented does not determine the attack type as the five attack types are merged into one set with two classes (benign–attacker). Instead, it only determines whether an attack exists or not.

So et al.^37^ trained two ML algorithms using a feature vector of six distinct features for BCS and BCM classification. The first algorithm used is the Support Vector Machine (SVM), while the second is the K-Nearest Neighbors (KNN) algorithm. The features included Location Plausibility (LP), Movement Plausibility (MP), and other features focused on calculating differences in the sender’s velocity and distance. Sharma and Liu^38^ extended this work by experimenting with more ML algorithms, including Ensemble Boosting (EB), Ensemble Voting (EV), Naïve Bayes (NB), and Random Forest (RF). They found that implementing plausibility checks increased detection performance for most types of attacks.

Ercan et al.^39^ employed two unique features, in addition to the regularly used features, to develop KNN and RF ML models for detecting the COP attack type. The first new feature determines the sender-receiver claimed distance, while the second one calculates the distance using an equation based on the RSSI and path loss. In their following research^40^, they employed KNN, RF, and ensemble learning ML models to examine various feature set combinations for BCS classification across varied traffic volumes. They added the difference between estimated and stated distances in BSMs in these combinations as another new feature.

In^41^, four derived features were used to train a federated machine learning solution for identifying position manipulation attacks in VANET. The federated learning method has several advantages, including enhanced privacy and the capability of implementing models on massive datasets without sharing the data between nodes and the central server. The performance obtained from this model was insufficient, and the authors attributed the limited performance of their models to the poor compatibility of the VeReMi dataset with federated learning approaches.

Kim et al.^42^ trained different models using Extreme Gradient Boosting (XGB), Multi-layer Perceptron (MLP), KNN, SVM, and RF algorithms to evaluate the performance of two different feature sets, named (Basic and Ext). In the “Basic” feature set, all features from two consecutive BSMs, sent by the same sender, are combined into a single feature set for each message. On the other hand, the “Ext” feature set comprises additional differential features beyond the “Basic” feature set. According to the results, using the “Ext” feature set enhanced the accuracy of all detectors.

The ML-based studies surveyed are summarized in Table 1. The table highlights the ML algorithms, the mode of classification, and the features used in each research. The comparison reveals that SVM, KNN, and RF are commonly used algorithms in recent studies. Moreover, it emphasizes the importance of utilizing derived features and plausibility checks to enhance the feature sets used for training ML models, rather than relying solely on the features of the BSMs.

**Table 1:** Summary of the surveyed ML-based studies.

Ref.	Algorithm	Mode	Features
So et al.^37^	SVM and KNN	BCS and BCM	LP
			MP
			The difference between the total and estimated distances using average speeds
			The difference between the estimated and average velocities
			The magnitude difference between the estimated and average velocities
Sharma and Liu^38^	EB, EV, SVM, NB, RF, and KNN	BCS and BCM	LP
			MP
			The difference between the total and estimated distances using average speeds
			The difference between the estimated and average velocities
			The magnitude difference between the estimated and average velocities
Ercan et al.^39^	KNN and RF	COP attack type	Sender-receiver distance
			RSSI
			Sender’s claimed location
			The variance in location between the latest and previous messages from the same sender
			Sender-receiver angle
			Calculated distance using RSSI
Ercan et al.^40^	Ensemble learning, KNN, and RF	BCS	The difference in the reported location between the most recent and previous messages from the same sender
			The variance in the location of the receiver between the latest and previous messages
			Sender-receiver angle
			Calculated distance using RSSI
			The discrepancy between the RSSI-based estimated distance and the distance derived from the message’s reported location
Uprety et al.^41^	Federated ML	BCS and BCM	The difference between the total and estimated distances using average velocities
			The difference between the estimated and average velocities
			The magnitude difference between the estimated and average velocities
Kim et al.^42^	KNN, SVM, RF, XGB, and MLP	BCM	All the features in the latest and previous messages
			MP
			The variance in claimed location between the latest and previous messages from the same sender
			The time variance between the latest and previous messages
			The Euclidean distance between the latest and previous messages

From the various experiments conducted in each reviewed research study —all of which utilized the VeReMi dataset to ensure consistent benchmarking conditions— the top-performing models were identified based on their average performance indicators. This consistency in dataset usage allows for a more meaningful and fair comparison of performance results across studies. Table 2 reviews the ML algorithm and performance outcomes of these models for BCS and BCM classification modes. For the CP attack, the Ensemble Boosting model by P. Sharma and Liu^38^ achieved the highest accuracy and F1-score with values 96.8% and 92.35%, respectively. For the COP attack, the KNN model by Ercan et al.^39^ demonstrated the best accuracy (95.01%) despite its lower F1-score (85.56%).

**Table 2:** Top-performing models for each research.

Paper	Algorithm	Attack type	Accuracy	Precision	Recall	F1-score
So et al.^37^	SVM	CP	0.9564	1	0.829	0.9065
		COP	0.7543	0.5729	0.1788	0.2725
		RP	0.9116	0.8149	0.886	0.849
		ROP	0.9177	0.8035	0.8755	0.838
		ES	0.8403	0.8162	0.4636	0.5913
		BCM	0.8838	0.8716	0.6515	0.7456
P. Sharma and Liu^38^	Ensemble Boosting	CP	0.968	1	0.8579	0.9235
	RF	COP	0.7795	0.2966	0.2829	0.2901
	Ensemble Boosting	RP	0.9713	0.9997	0.8801	0.936
	Ensemble Boosting	ROP	0.9704	0.9218	0.7395	0.8207
	SVM	ES	0.8403	0.8162	0.4636	0.5913
	RF	BCM	0.9082	0.9917	0.2857	0.4436
Ercan et al.^39^	KNN	COP	0.9501	0.8552	0.8559	0.8556
Ercan et al.^40^	RF	CP	0.8566	–	–	0.912
		COP	0.868	–	–	0.9186
		RP	0.8466	–	–	0.9063
		ROP	0.841	–	–	0.8693
		ES	0.8663	–	–	0.916
Uprety et al.^41^	Federated ML	CP	–	0.9455	0.81	0.8725
		COP	–	0.6758	0.63	0.6521
		RP	–	0.8675	0.61	0.7163
		ROP	–	0.8674	0.63	0.7299
		ES	–	0.9294	0.73	0.8177
		BCM	0.7990	–	–	–
Kim et al.^42^	MLP	BCM	0.9910	1.0000	0.9830	0.9910

In the case of RP attack detection, the Ensemble Boosting algorithm by P. Sharma and Liu^38^ outperformed the other models, achieving 97.13% accuracy and a 93.6% F1-score. For the ROP attack, the Ensemble Boosting algorithm again performed well, with an accuracy of 97.04% and an F1-score of 82.07%. In detecting the ES attack, the RF model by Ercan et al.^40^ achieved the highest performance, with an accuracy of 86. 63%, and an F1-score of 91.6%.

Finally, for the BCM attack, the MLP model introduced by Kim et al.^42^ demonstrated strong performance, achieving 99.10% in both accuracy and F1-score, but it was built on a centralized detection approach where data from multiple vehicles is collected and analyzed in a centralized manner. The main limitation of this approach is the reliance on the existence of Roadside Units (RSUs) along all roads to identify malicious nodes, which is not guaranteed in the VANET environment. Moreover, despite the high reported performance, the potential for slight enhancements should not be overlooked, as small improvements in detection accuracy can have a significant impact on the reliability and safety of VANET applications that directly affect human lives.

The findings from the review indicate that specific models, particularly those employing ensemble methods or integrating additional plausibility checks and derived features, demonstrate high performance in specific scenarios. However, their performance remains limited, especially when considering the critical safety risks posed to passengers if attacks are not accurately detected and identified.

## Proposed model

**Fig. 1:**
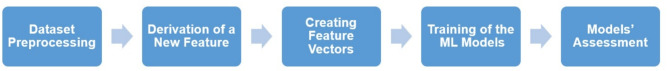
Implementation phases of the proposed model.

In this section, we implement and test several ML-based models using the VeReMi dataset. The implementation process has been accomplished in five phases, as summarized in Fig. 1. First, the dataset was prepared to be compatible with ML training. A new derived feature based on the received message’s RSSI was introduced in the next phase. Then, the new feature is added to a set of commonly used features to create three feature set combinations. In the fourth phase, four ML algorithms were trained using the three feature sets. Finally, the efficiency of all models was evaluated to select the best model.

### Dataset preprocessing

The VeReMi dataset comprises 19 features distributed between the message logs for every simulated node and a file that specifies the actual behavior of each simulated node (ground truth file). The message logs include the reception time, the claimed location and speed of the sender in the X, Y, and Z directions, the claimed sending time, the ID of the sender and the message, and the RSSI. Conversely, the other features added by the ground truth file are the sender’s true location and speed prior to manipulation, the actual transmission time, and the attack type.

Additionally, the dataset includes five simulation repetitions, each with a set of separate sub-datasets for each attack type, at three different traffic and attack densities. These repetitions, produced using varying random seeds, offer multiple simulation runs and message logs.

Thus, before model training, several preprocessing steps were applied. First, all message logs were consolidated into a single dataset representing the complete simulation. Empty or unused features were removed, and each message was associated with its corresponding ground truth information. In addition, a merged dataset was constructed by combining all attack variants into a binary classification setting, consisting of one class representing malicious messages and another representing legitimate messages.

The categorical labels corresponding to attack types were encoded using integer label encoding prior to model training. All remaining features are continuous numerical values derived directly from the BSMs or computed during preprocessing; therefore, feature normalization or scaling was not applied.

### Proposed feature

In this phase, a new feature that relies on the RSSI of the received BSM is derived to enhance the detection of position falsification attacks. RSSI represents the calculated power level of a received radio signal, expressed in decibels relative to a milliwatt (dBm). It is a key metric in wireless communication systems that can be used to measure the quality of a signal and estimate the distance between a transmitter and a receiver^43^. However, directly using the RSSI for location estimation can lead to inaccurate outcomes due to external factors like signal fading and environmental obstacles^44^.

Rather than using RSSI as an independent feature or directly calculating the sender-receiver distance, the proposed feature correlates RSSI values with their confidence intervals at a given distance range. This concept is inspired by the work of^30^, which introduced RSSI-based plausibility checks to detect location spoofing using predefined RSSI confidence ranges. However, their approach did not incorporate ML techniques or other BSM features, relying solely on predefined thresholds. Additionally, their method employed a fixed confidence level of 99.7% in all calculations. In contrast, the proposed method assigns a location reliability score to each message using three different confidence levels, reflecting the degree of trust in the sender’s claimed location. This score is not a standalone classification mechanism but rather an enhancement that, when integrated with other key features, strengthens the detection of position spoofing attacks through ML techniques.

Initially, the RSSI confidence intervals were calculated in each distance range. To achieve this, a dataset version was created that contained only legitimate messages. Then, the sender-receiver distance ($d_s$) for each message was computed using the Euclidean distance. The Euclidean distance can be calculated using the following formula:

1 $$\begin{aligned} d_s = \sqrt{(x_s - x_r)^2 + (y_s - y_r)^2} \end{aligned}$$

where $x_s$ and $y_s$ are the sender coordinates and $x_r$ and $y_r$ are the receiver coordinates, in the X and Y directions^45^. Then, the dataset was segmented into several segments using the computed distance. For each segment, i.e., distance range, the RSSI distribution was used to calculate the RSSI confidence intervals based on its mean and variance at that distance range.

The confidence interval of sample statistics reflects the values within which the valid population parameter is expected to lie, given a specified confidence level or probability. The probability assigned to this range is the confidence level, while the higher and lower values are referred to as confidence boundaries. The following equation can identify these boundaries:

2 $$\begin{aligned} B = m \pm z \times \frac{sd}{\sqrt{n}} \end{aligned}$$

where B is the upper or lower boundary, m is the sample’s mean, z is a value that depends on the specified confidence level, sd is the sample’s standard deviation, and n is the sample size^46^. The confidence intervals vary according to the chosen confidence level, as a higher confidence level results in a broader range.

**Fig. 2:**
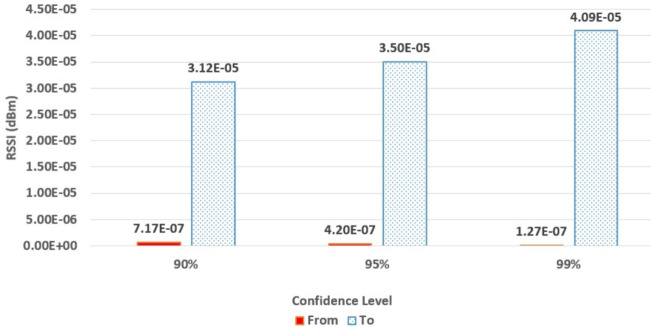
RSSI confidence intervals at (2 to 4) units Euclidean distance between sender and receiver.

Thus, instead of using a single confidence level, the RSSI confidence intervals for each distance segment were calculated at 90%, 95%, and 99% confidence levels. Figure 2 illustrates the RSSI confidence boundaries when the sender-receiver Euclidean distance ranges from 2 to 4 units. Then, all results were stored in a dictionary that associates each distance range with its respective RSSI confidence intervals. Table 3 provides a sample of the data within the created dictionary. Later, this precomputed dictionary can be published to vehicles via RSUs or by equipping cars with predefined dictionaries, to enable distributed deployment of the detection model.

**Table 3:** The created distance-to-RSSI dictionary.

Distance			RSSI confidence range	
From	To	Confidence level	From	To
0	2	90%	1.76e−07	2.54e−05
0	2	95%	1.72e−07	5.70e−05
0	2	99%	1.47e−07	7.61e−05
2	4	90%	7.17e−07	3.12e−05
2	4	95%	4.20e−07	3.50e−05
2	4	99%	1.27e−07	4.09e−05
			...	
> 700		90%	1.28e−09	1.52e−09
> 700		95%	1.27e−09	1.56e−09
> 700		99%	1.27e−09	1.62e−09

The location of a received message can be classified as spoofed or not by checking whether the RSSI value falls within the confidence intervals associated with the claimed distance. To measure the effectiveness of this classification approach, the true positives, true negatives, false positives, and false negatives were calculated for the classification of BCM using confidence intervals at the three confidence levels. The performance results, as shown in Table 4, indicate that using a 90% confidence level yields the lowest precision and the highest recall, due to an increase in the count of false positives and a reduction in the count of false negatives. On the other hand, the 99% confidence level, which uses a wider interval, results in the highest precision and the lowest recall due to the reduced number of false positives and the increased number of false negatives.

**Table 4:** Performance results of using different confidence levels for BCM classification.

Level	Accuracy	Precision	Recall	F1-score
90%	0.8871	0.042	0.6052	0.0785
95%	0.9416	0.0663	0.4844	0.1166
99%	0.9845	0.1725	0.2474	0.2033

**Fig. 3:**
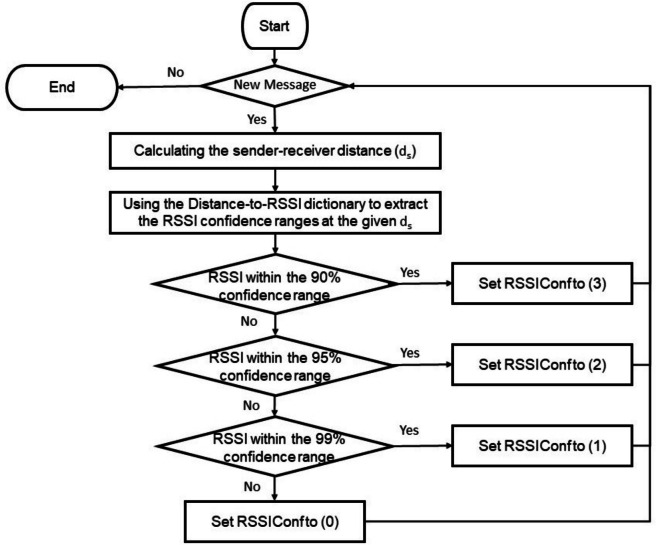
The calculation process of the proposed feature.

Building on these findings, a new feature called “RSSIConf” was developed to break the dependency on a single confidence interval. In this feature, the RSSI value in each BSM is compared to the confidence intervals at the three distinct levels corresponding to the stated sender-receiver distance. The RSSIConf feature is then assigned a value of 3 if the RSSI is within the 90% confidence interval, 2 for the 95% interval, and 1 for the 99% interval. If the RSSI does not fall within any of these intervals, RSSIConf is set to 0, suggesting a higher risk of data fabrication. This feature derivation process was repeated for all dataset rows to add the new feature to the training dataset, as explained in Fig. 3.

It is important to note that the computationally intensive task of generating the RSSI confidence intervals and constructing the distance-to-RSSI dictionary is performed offline. Once constructed, the dictionary can be stored locally or distributed to vehicles via roadside units. During online operation, the RSSIConf feature calculation requires only a simple dictionary lookup to retrieve the corresponding confidence intervals for the claimed sender–receiver distance, followed by a small number of comparison operations. As a result, the runtime complexity of RSSIConf computation is constant time, introducing minimal computational overhead and enabling efficient real-time deployment in VANET environments.

### Creating feature vectors

In this phase, we combine the newly derived feature with commonly selected and derived features to create several feature sets. This approach aims to enhance the model’s reliability, as using all of the existing BSM features to implement ML-based MDSs without incorporating feature engineering techniques can lead to potential bias and inflated results that significantly drop when models are assessed using simulations distinct from those employed during development^47^. Pearson’s correlation coefficient (PCC) technique was employed to identify the most effective BSM features for attack classification. The PCC quantifies the degree of similarity and the strength of the relationship between two variables, reflecting their level of dependence^48^. By performing correlation analysis on the features, it becomes possible to detect redundancies within the dataset. Additionally, examining the relationship between features and classification labels helps identify which features have a significant influence on the classification process^49^. The analysis results suggest a strong correlation between the sender’s location and speed along the X and Y coordinates, as well as the identification of location manipulation attacks.

PCC was employed in this analysis instead of other ranking-based methods, such as Spearman’s rank correlation (SPC), as PCC is widely used in VANET feature selection studies for identifying redundant and highly dependent features prior to machine learning training. The objective of the feature selection stage in this work is not only to examine the relationships between features and the classification labels, but also to reduce feature redundancy among the selected attributes. Since the considered BSM features represent continuous-valued physical measurements, PCC provides an effective and sufficient measure for detecting linear dependencies and redundancy among such features.

Finally, the selected feature set was enhanced by integrating derived features that have shown strong performance in previous studies. The survey identified two frequently utilized derived features in high-performing models. These features include the variance in the sender’s reported location between consecutive BSMs and the sender-receiver distance. Additionally, other differential features were considered, such as variations in the sender’s speed and transmission time between consecutive BSMs, as well as the sender’s most recent broadcast position and speed. These features were organized into three distinct feature vectors (FV1, FV2, and FV3), as outlined in Table 5, where features marked with the symbol “*” denote the features included in the corresponding feature vector and were computed prior to model training.

**Table 5:** The features of FV1, FV2, and FV3.

Feature	FV1	FV2	FV3
RSSIConf	*	*	*
Sender’s location	*	*	*
Sender’s speed	*	*	*
The sender-receiver distance	*	*	*
The variance in claimed location between the sender’s latest and previous messages		*	*
The variance in claimed speed between the sender’s latest and previous messages		*	*
The variance in transmission time between the sender’s latest and previous messages		*	*
The previous message’s location			*
The previous message’s speed			*

### Training the models

In the training phase, multiple BCS and BCM classification models were developed by training RF, KNN, XGB, and MLP ML algorithms using the three created feature vectors. RF and KNN were chosen because they are often used in high-performing models, while XGB was employed because of its high performance and robustness to overfitting^50^. In addition, the MLP algorithm was chosen to experiment with a simple neural network architecture because of its outstanding efficiency in BCM classification, as presented by Kim et al.^42^.

All experiments were conducted on a Windows-based system using Python 3.7.6 within the Jupyter Notebook environment provided as part of the Anaconda Distribution (Anaconda3-2020.02) (https://www.anaconda.com/download). The ML models were implemented using the scikit-learn and XGBoost Python libraries. For each algorithm, key hyperparameters were selected based on a trade-off between detection performance and computational efficiency, with particular consideration for real-time VANET deployment constraints. For instance, since RF and XGB are ensemble learning techniques, it is necessary to initialize the count of decision trees built during training. This number is a critical parameter that influences both generalization performance and computational cost. In this study, the number of trees was set to 50. Preliminary experiments showed that increasing the number of trees beyond this value resulted in negligible performance improvements while substantially increasing training and inference time. Consequently, 50 trees were selected as a balanced configuration that provides stable performance without unnecessary computational overhead.

The KNN classifier assigns class labels based on the majority class of the training set’s nearest neighbors, where ‘k’ is the count of neighbors used in the classification. The value of ‘k’ was set to 3, as using small values of ‘k’ reduces the number of distance calculations and sorting operations required during inference, which is desirable in time-sensitive VANET applications. Initial testing indicated that larger k values increased computational cost and slightly smoothed decision boundaries without yielding consistent performance gains.

For the MLP classifier, the network architecture was adopted from the configuration reported in^42^, which demonstrated strong performance for similar misbehavior detection tasks. The architecture consists of three hidden layers, each with eight neurons, using “ReLU,” “ReLU,” and “Sigmoid” activation functions, respectively. All remaining hyperparameters for the evaluated models were kept at their default values to avoid unnecessary complexity.

Overall, initial experimental evaluations indicated that moderate variations around the selected hyperparameter values did not lead to significant changes in classification performance, whereas larger deviations mainly increased computational cost without improving detection accuracy.

### Models’ assessment

In the assessment phase, the performance of all pre-trained schemes was used to select the model with the highest performance. Four Key Performance Indicators (KPIs) were employed to assess the models’ efficiency. The first KPI is accuracy, which measures the model’s ability to identify positive and negative inputs. Thus, accuracy increases when true positive and true negative rates are higher. However, focusing on accuracy may be insufficient for assessing MDS performance, particularly with imbalanced datasets^51^. Precision, the second KPI used, is the proportion of correctly identified positive samples to the total number of predicted positive samples. The third measure, recall, assesses the system’s ability to determine actual positive samples accurately. The last KPI is the F1-score, which provides a balanced statistic that considers both false positives and negatives^38^.

The models were tested using a randomly chosen 30% subset of each dataset. Tables 6, 7, 8, 9, 10 and 11 present the performance results obtained for all tested models. The outcomes show that the RF model achieved 100% measures for the CP attack detection when employing any of the three feature vectors (FV1, FV2, or FV3). The XGB model achieved the same perfect performance using FV1 and a near-perfect performance using FV2 and FV3. In the COP attack, both RF and XGB models achieved over 99.9% performance employing any of the three feature vectors. However, the XGB models showed a very slight enhancement over the RF models.

**Table 6:** Performance results in the CP attack detection.

Algorithm	Features	Accuracy	Precision	Recall	F1-score
RF	FV1	1	1	1	1
	FV2	1	1	1	1
	FV3	1	1	1	1
KNN	FV1	0.9994	0.999	0.9996	0.9993
	FV2	0.9997	0.9995	0.9998	0.9996
	FV3	0.9998	0.9996	0.9998	0.9997
MLP	FV1	0.9923	0.992	0.9884	0.9902
	FV2	0.999	0.9983	0.9991	0.9987
	FV3	0.9971	0.9948	0.9979	0.9963
XGB	FV1	1	1	1	1
	FV2	0.9999	0.9999	0.9999	0.9999
	FV3	0.9999	0.9999	0.9999	0.9999

**Table 7:** Performance results in the COP attack detection.

Algorithm	Features	Accuracy	Precision	Recall	F1-score
RF	FV1	0.9992	0.9992	0.9989	0.999
	FV2	0.999	0.9991	0.9985	0.9988
	FV3	0.9994	0.9994	0.9991	0.9992
KNN	FV1	0.998	0.9978	0.997	0.9974
	FV2	0.9971	0.997	0.9959	0.9964
	FV3	0.9976	0.9973	0.9966	0.997
MLP	FV1	0.9619	0.9486	0.9572	0.9528
	FV2	0.9626	0.9507	0.9566	0.9536
	FV3	0.9604	0.9503	0.9508	0.9506
XGB	FV1	0.9996	0.9995	0.9994	0.9995
	FV2	0.9995	0.9995	0.9993	0.9994
	FV3	0.9994	0.9994	0.9991	0.9993

**Table 8:** Performance results in the RP attack detection.

Algorithm	Features	Accuracy	Precision	Recall	F1-score
RF	FV1	0.9997	0.9997	0.9996	0.9997
	FV2	0.9999	0.9999	0.9999	0.9999
	FV3	0.9999	0.9999	0.9998	0.9999
KNN	FV1	0.9996	0.9996	0.9994	0.9995
	FV2	0.9998	0.9998	0.9997	0.9997
	FV3	0.9998	0.9998	0.9997	0.9997
MLP	FV1	0.9975	0.9982	0.9962	0.9972
	FV2	0.9996	0.9997	0.9994	0.9995
	FV3	0.9996	0.9997	0.9994	0.9995
XGB	FV1	0.9997	0.9998	0.9996	0.9997
	FV2	0.9999	0.9999	0.9998	0.9999
	FV3	0.9999	0.9999	0.9998	0.9999

**Table 9:** Performance results in the ROP attack detection.

Algorithm	Features	Accuracy	Precision	Recall	F1-score
RF	FV1	0.9917	0.9921	0.9885	0.9902
	FV2	0.997	0.9975	0.9954	0.9964
	FV3	0.9972	0.9977	0.9958	0.9967
KNN	FV1	0.9722	0.9774	0.9578	0.9668
	FV2	0.9897	0.9903	0.9854	0.9878
	FV3	0.9908	0.992	0.9862	0.989
MLP	FV1	0.8619	0.8543	0.8138	0.8296
	FV2	0.9725	0.98	0.9554	0.9666
	FV3	0.9716	0.9795	0.9537	0.9654
XGB	FV1	0.9764	0.9802	0.9648	0.9720
	FV2	0.9958	0.9967	0.9935	0.9951
	FV3	0.9959	0.9967	0.9935	0.9951

**Table 10:** Performance results in the ES attack detection.

Algorithm	Features	Accuracy	Precision	Recall	F1-score
RF	FV1	0.9982	0.9979	0.9977	0.9978
	FV2	0.9989	0.999	0.9984	0.9987
	FV3	0.999	0.9991	0.9986	0.9988
KNN	FV1	0.9531	0.9407	0.9483	0.9444
	FV2	0.9714	0.9675	0.9642	0.9658
	FV3	0.9731	0.9693	0.9664	0.9679
MLP	FV1	0.8499	0.8222	0.814	0.8179
	FV2	0.942	0.9418	0.9184	0.929
	FV3	0.9416	0.9565	0.9266	0.9058
XGB	FV1	0.9860	0.9869	0.9796	0.9832
	FV2	0.9923	0.9937	0.9879	0.9908
	FV3	0.9912	0.9930	0.9860	0.9894

**Table 11:** Performance results in the BCM classification.

Algorithm	Features	Accuracy	Precision	Recall	F1-score
RF	FV1	0.9956	0.9957	0.9953	0.9955
	FV2	0.9986	0.9985	0.9984	0.9984
	FV3	0.9986	0.9986	0.9984	0.9985
KNN	FV1	0.9726	0.9735	0.9704	0.9718
	FV2	0.9836	0.9838	0.9797	0.9817
	FV3	0.9836	0.9838	0.9797	0.9817
MLP	FV1	0.8795	0.8791	0.8731	0.8757
	FV2	0.8943	0.9061	0.8588	0.8762
	FV3	0.8846	0.9019	0.8431	0.863
XGB	FV1	0.9711	0.9742	0.9671	0.9702
	FV2	0.9764	0.9802	0.9676	0.9735
	FV3	0.9764	0.9802	0.9676	0.9735

For the RP attack, both RF and XGB models achieved over 99.9% performance employing FV2 and FV3. However, the RF model with FV2 (RF-FV2) outperformed the others, achieving the best results among all tested models with a very slight increase in recall percentage. Furthermore, in detecting ROP and ES attacks, the RF-FV3 models outperformed other models. For example, in the ROP attack, the RF-FV3 model achieved a detection accuracy of 99.72% and an F1-score of 99.67%. Similarly, the achieved accuracy and F1-score were 99.9% and 99.88% in the ES attack detection. When used for BCM classification, the RF-FV3 model achieved the highest performance, with 99.86% accuracy and a 99.85% F1-score.

**Fig. 4:**
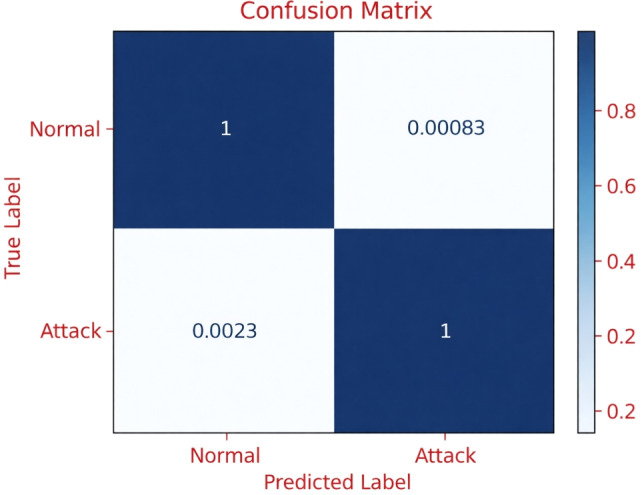
The confusion matrix of RF-FV2 in BCM classification.

**Fig. 5:**
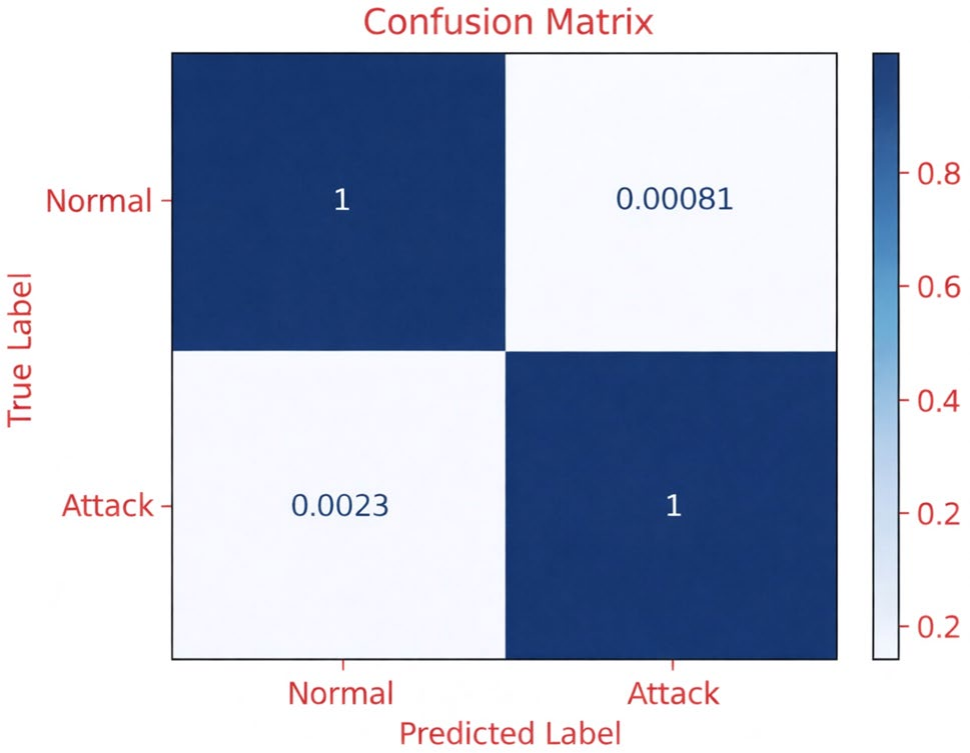
The confusion matrix of RF-FV3 in BCM classification.

The research findings show that although the XGB-FV2 and XGB-FV3 models had almost similar performance to the RF-FV2 and RF-FV3 models in detecting CP, COP, and RP attacks, they were less efficient in ROP, ES, and BCM classification. These results also indicate that employing RF with either FV2 or FV3 is almost equally successful at identifying the five attack types, including BCM categorization, with only a slight performance increase provided by RF-FV3 in ROP, ES, and BCM classification, which does not exceed 0.02%. Thus, both RF-FV2 and RF-FV3 can be considered the top models among the trained models. This observation is further supported by the confusion matrices obtained for the RF-FV2 and RF-FV3 models in the BCM classification. As illustrated in Figures 4 and 5, both models achieve very high true positive and true negative rates, with only marginal misclassification between benign and attack classes. Confusion matrix analysis is presented for FV2 and FV3 only, as FV1 consistently exhibited lower performance across all evaluated metrics and attack types; therefore, its confusion matrix was omitted to maintain focus on the most competitive feature sets.

**Fig. 6:**
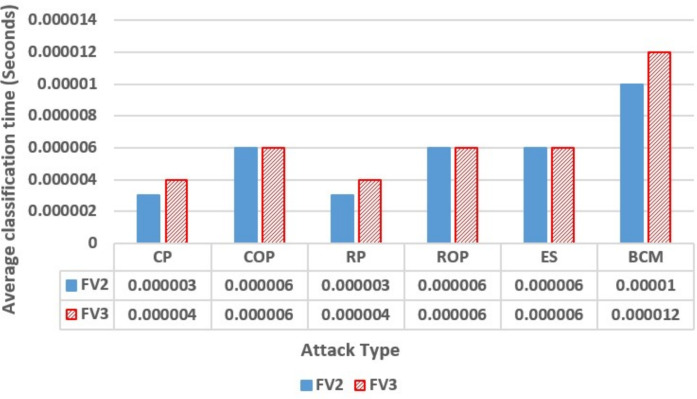
The average time the RF models took from message reception to output classification using FV2 and FV3.

The processing time of a pre-trained model, measured from message reception to output classification, was included in the performance comparison of the models. This metric provides an estimate of the computational efficiency of the models during deployment and is calculated by dividing the total testing time by the number of samples processed. Figure 6 presents a comparative analysis of the average classification time for the RF-FV2 and RF-FV3 models across individual attack types as well as the BCM scenario.

For CP and RP attacks, RF-FV3 exhibits a slightly higher classification time than RF-FV2, which can be attributed to the additional feature processing introduced by the expanded feature set. In contrast, for COP, ROP, and ES attacks, both models demonstrate nearly identical execution times, indicating that the added features in FV3 do not noticeably impact runtime for these scenarios. In the BCM case, the execution-time difference becomes more pronounced, with RF-FV3 performing up to 25% slower than RF-FV2. This behavior can be explained by the fact that the BCM dataset encompasses a larger and more diverse set of samples, which increases the number of feature evaluations required per classification. Overall, these results indicate that RF-FV2 incurs lower computational cost.

In real VANET environments, multiple position falsification attacks can occur at the same time, so deploying multiple specialized detection models is a realistic issue in terms of feasibility. While training individual models for every attack might provide worthwhile insights, deploying multiple models simultaneously would incur greater computational cost and undermine efficiency in real-time applications. Therefore, the primary objective of training individual models within this study was to facilitate a comparison of how the selected sets of features and ML algorithms performed on various attack types, which is a commonly used methodology in related literature.

Based on the above evaluation, the RF-FV2 model was selected as the primary detection model because it provides a favorable trade-off between detection accuracy and computational efficiency, as reflected in its consistently strong performance across all evaluated attack types and its lower inference latency compared to alternative models. In addition, RF effectively handles heterogeneous and correlated features with limited sensitivity to parameter tuning, which contributes to stable generalization performance and makes it suitable for real-time deployment in dynamic VANET environments^52^. Moreover, RF-FV2 was trained and tested under the BCM classification scenario, which incorporates all five attack categories, and achieved high detection performance, making it a more representative and reliable choice for deployment in realistic VANET settings.

## Evaluation and deployment

The evaluation process of the proposed model is separated into two steps. Firstly, the performance of the chosen model (RF-FV2) was compared to that of the highest-performing models in the literature. Then, the effectiveness and reliability of the proposed feature were analyzed.

### Performance comparison

In this step, the proposed model’s efficiency was compared to that of the best-performing ML-based models in each reviewed paper. All of the compared models were implemented using the VeReMi dataset to eliminate potential biases during the assessment process. Tables 12 and 13 compare the (RF-FV2) model to existing literature models regarding accuracy and F1-score, respectively.

**Table 12:** Accuracy of the proposed model and existing models.

Model	CP	COP	RP	ROP	ES	BCM
RF-FV2	1.0000	0.9990	0.9999	0.9970	0.9989	0.9986
So et al.^37^	0.9564	0.7543	0.9116	0.9177	0.8403	0.8838
Sharma and Liu^38^	0.9680	0.7795	0.9713	0.9704	0.8403	0.9082
Ercan et al.^39^	–	0.9501	–	–	–	–
Ercan et al.^40^	0.8566	0.8680	0.8466	0.8410	0.8663	–
Uprety et al.^41^	–	–	–	–	–	0.7990
Kim et al.^42^	–	–	–	–	–	0.9910

**Table 13:** F1-score of the proposed model and existing models.

Model	CP	COP	RP	ROP	ES	BCM
RF-FV2	1.0000	0.9988	0.9999	0.9964	0.9987	0.9984
So et al.^37^	0.9065	0.2725	0.8490	0.8380	0.5913	0.7456
Sharma and Liu^38^	0.9235	0.2901	0.9360	0.8207	0.5913	0.4436
Ercan et al.^39^	–	0.8556	–	–	–	–
Ercan et al.^40^	0.9120	0.9186	0.9063	0.8693	0.9160	–
Uprety et al.^41^	0.8725	0.6521	0.7163	0.7299	0.8177	–
Kim et al.^42^	–	–	–	–	–	0.9910

Analyzing the performance results, the RF-FV2 model consistently demonstrates higher performance than the compared models across multiple attack types under the evaluated simulation scenarios, or at least performs similarly in some cases, as follows:

- CP attack: This attack is less challenging to identify as the attacker broadcasts a static, false location that deviates significantly from typical behavior, in which the vehicle’s position shifts from message to message. The proposed model outperformed the best model among the compared ones by a 3.2% increase in accuracy and a 7.65% improvement in F1-score.
- COP attack: The COP attack involves an attacker broadcasting the real location, which has been modified by a constant offset value. Similar to the CP attack, the RF-FV2 model demonstrates strong detection performance for the COP attack within the evaluated simulation scenarios, achieving accuracy and F1-score improvements of 4.89% and 8.02%, respectively, compared to the best-performing reference model.
- RP and ROP attacks: The RP attack includes the attacker broadcasting random, false locations, while in the ROP attack, the attacker offsets the actual position by a random value, making identification more difficult owing to the lack of typical patterns. In the RP attack detection, the RF-FV2 model outperformed the leading model by 2.86% and 6.39% in accuracy and F1-score, respectively. Furthermore, in the ROP attack, the proposed model improved accuracy by up to 2.66% and F1-score by 12.71% over the best model.
- ES attack: The ES attack involves the attacker mimicking regular driving activity before falsely reporting a halt. The RF-FV2 performance in the ES attack outperformed the compared top model by 13.26% and 8.27% in accuracy and F1-score, respectively.
- BCM attack: In this classification type, the enhancement introduced by the RF-FV2 model is 0.76% and 0.74% in accuracy and F1-score over the top models.

The results reveal that the proposed RF-FV2 model achieves perfect accuracy and F1-score for the CP attack under the evaluated VeReMi simulation settings. Although such performance may appear unusually high, it can be explained by the nature of the CP attack as well as the adopted feature set. In this attack type, the attacker consistently broadcasts a falsified but fixed position throughout the simulation, resulting in a persistent discrepancy between the claimed location and the actual physical distance inferred from the received signal strength. The RSSIConf feature is specifically designed to capture this inconsistency by comparing the measured RSSI against confidence intervals corresponding to the claimed sender–receiver distance, making CP attacks particularly distinguishable. In addition, the structure of the VeReMi dataset contributes to this outcome. In VANET simulations, the same transmitted message may be received by multiple neighboring vehicles, leading to repeated message instances in the dataset with variations in certain features such as sender–receiver distance and RSSI values. This behavior may amplify performance metrics to some extent; so, the objective of this evaluation is to assess the effectiveness of the proposed approach relative to existing detection schemes that rely on the same dataset and evaluation technique.

Moreover, while COP and ES attacks may appear similar to normal driving behavior, they can still be detected by the proposed model using the RSSIConf feature. Even if the vehicle’s movement looks realistic, the mismatch between the signal and the claimed position can reveal falsified behavior. This is reflected in the improved detection results, with the model achieving up to 4.89% and 8.02% increases in accuracy and F1-score for the COP attack, and 13.26% and 8.27% for the ES attack. Although the model also might show smaller improvements in other attack types, the obtained enhancements are significant in VANET environments where even small gains in detection performance can help prevent safety-critical decisions based on false information.

Although computational overhead is a key factor in evaluating misbehavior detection solutions, a direct comparison with existing models was not feasible, as the reviewed literature did not report their runtime complexity or latency performance. To address this limitation, we conducted an internal latency assessment by measuring the approximate average classification time of each trained model, as illustrated previously in Figure 6, where the RF-FV2 model demonstrated a competitive inference latency, making it well-suited for time-sensitive VANET applications. While a detailed theoretical complexity analysis was not included, this empirical evaluation supports the practical efficiency of the proposed model in environments where fast and reliable decision-making is critical.

### Proposed feature evaluation

A modified version of the RF-FV2 model, called RF-FV2’, was implemented to assess the impact of the introduced RSSIConf feature. The RF-FV2’ model was trained using the same dataset, ML algorithm, and feature set as RF-FV2, except that RSSIConf was excluded. Then, both models were evaluated on the same dataset to compare their performance.

According to the surveyed studies, datasets are typically divided into separate training and testing subsets to avoid biased evaluation, as using the same data for both phases can lead to overfitting. Overfitting occurs when a model becomes overly specialized in capturing patterns present in the training data, resulting in poor generalization to unseen data. In VANET datasets, however, this issue can persist even after a conventional train–test split, because the same transmitted message may be received by multiple neighboring vehicles, creating overlap between the training and testing subsets and increasing the risk of information leakage. Consequently, model performance measured under such conditions may appear artificially high.

To mitigate this hidden form of overfitting and improve evaluation diversity, the performance of the two models ( RF-FV2 and RF-FV2’) was evaluated using an entirely different simulation repetition provided by the VeReMi dataset. The VeReMi dataset includes five independent repetitions generated using different random seeds, each producing a distinct set of message logs. Evaluating models on a separate repetition ensures that all classified messages are unseen during training, thereby providing a more reliable assessment of generalization performance. Similar to the previous approach, an additional dataset was created using the selected repetition, in addition to the five datasets for each attack type. The supplementary dataset integrates all attack types, enabling the assessment of the models within the BCM classification.

**Fig. 7:**
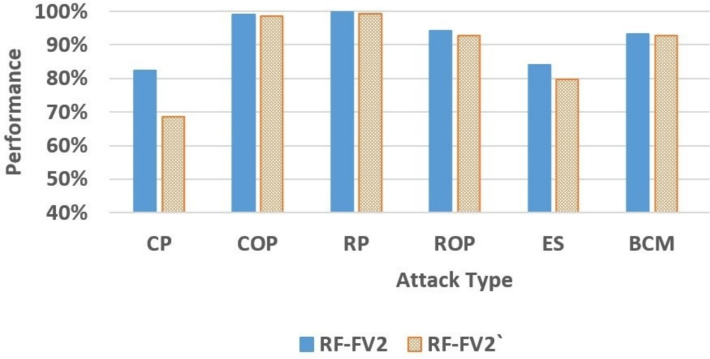
The accuracy comparison between RF-FV2 and RF-FV2’.

**Fig. 8:**
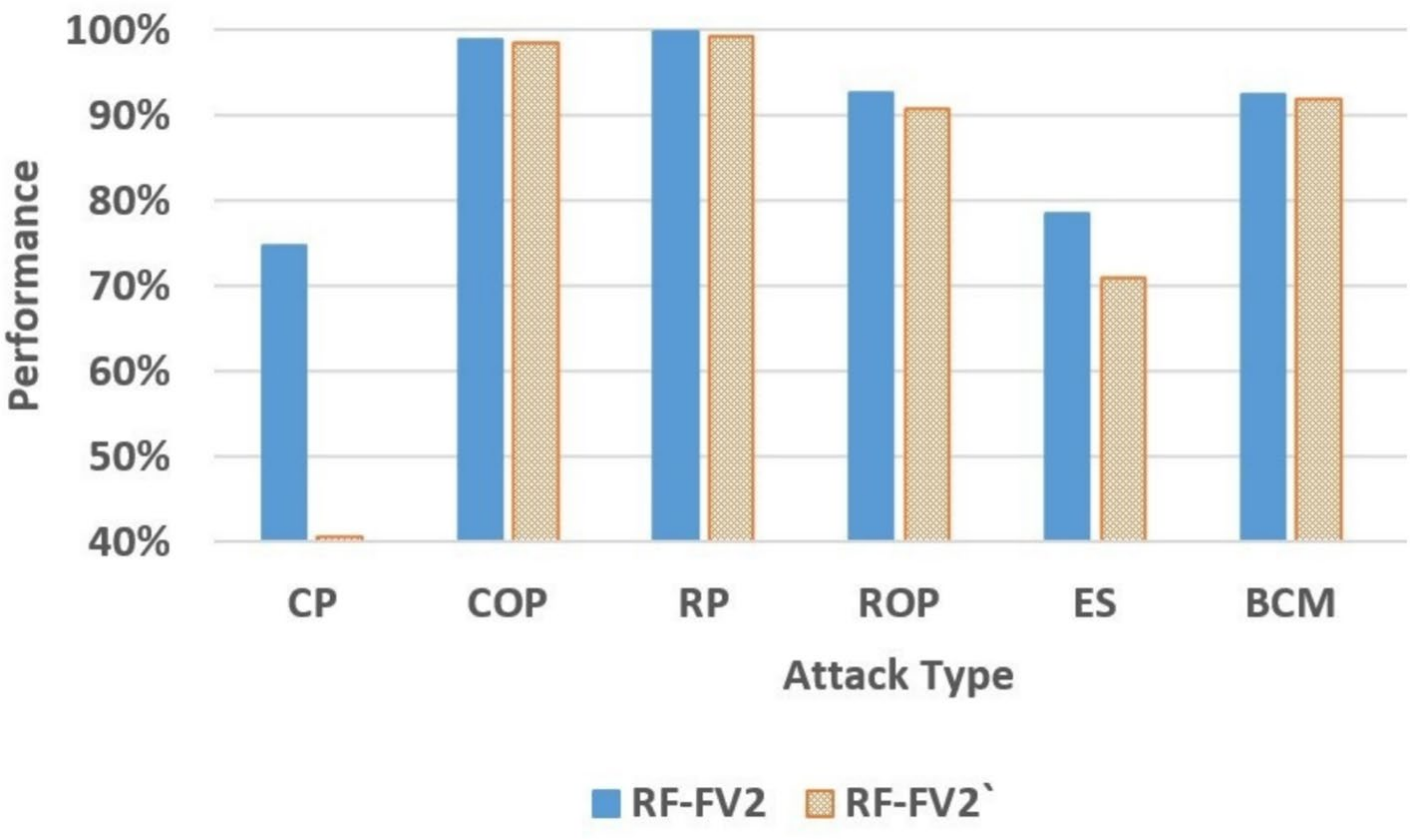
The F1-score comparison between RF-FV2 and RF-FV2’.

The pre-trained RF-FV2 and RF-FV2’ models were subsequently applied to classify the messages in the selected datasets. Table 14 summarizes the comparative performance improvements made by the RF-FV2 over the RF-FV2’ across the different attack and classification types. The percentage enhancements in the table show how the RSSIConf feature increased the detection capabilities of RF-FV2. Furthermore, Figs. 7 and 8 provide a graphical comparison of the two models’ performance, illustrating that although most models experience a reduction in performance when evaluated on a new simulation, this degradation is consistently less pronounced for RF-FV2 than for RF-FV2’. These results indicate that the inclusion of the RSSIConf feature enhances model robustness and generalization under unseen simulation conditions, reducing the impact of performance inflation caused by data overlap.

**Table 14:** The performance improvements made by the RF-FV2 over the RF-FV2’.

Attack	Accuracy	Precision	Recall	F1-score
CP	20.16%	162.06%	43.82%	83.89%
COP	0.33%	0.25%	0.51%	0.39%
RP	0.50%	0.95%	0.40%	0.68%
ROP	1.63%	2.13%	2.19%	2.16%
ES	5.23%	4.99%	10.50%	10.49%
BCM	0.57%	0.59%	0.71%	0.64%

The accuracy metric shows that the RSSIConf feature considerably enhanced the model’s performance in the CP attack by 20.16% over RF-FV2’. This is a significant improvement, demonstrating that RSSIConf is crucial for improving the detection of the CP attack. Other attacks, such as ES and ROP, showed improvements of 5.23% and 1.63%, respectively. Although the accuracy enhancement for COP, RP, and BCM attacks was minor, varying from 0.33% to 0.57%, they still highlight the usefulness of including RSSIConf in the feature vector.

Furthermore, analyzing the F1-score results for each attack type revealed that the CP attack achieved an improvement of 83.89% in the F1-score. Similarly, for the ES attack, the F1-score increased by 10.49%. For the other attack types (COP, RP, ROP, and BCM), the F1-score improved by 0.39% to 2.16%. The improvement in the F1-score reflects the enhancement introduced in the precision and recall of the models’ detection. Additionally, it demonstrates that the RSSIConf feature enables the model to achieve more balanced and reliable detection across various attack types.

In addition to the performance comparison between the two models, the McNemar test was used to ensure that using the RSSIConf feature introduced a statistically significant difference between them. In that case, the McNemar test is based on the contingency table of the differences in the classification between the two models^53^. For two models X and Y that were tested using the same dataset, the contingency table consists of four cells as follows:

- Cell a: the count of correctly classified samples by both X and Y models.
- Cell b: the count of samples that were correctly classified by model X and misclassified by model Y.
- Cell c: the count of samples that were correctly classified by model Y and misclassified by model X.
- Cell d: the count of misclassified samples by both X and Y models.

Then, the test statistic focuses on the b and c values, and its value is computed as follows:

3 $$\begin{aligned} \chi ^2 = \frac{(b-c)^{2}}{b+c} \end{aligned}$$

Based on the test statistics, the p-value is calculated, and if it is less than or equal to 0.05, the two models exhibit a statistically significant difference; otherwise, there is no significant difference between them^54^.

Table 15 shows the contingency table of the classification outcomes of RF-FV2 and RF-FV2’ models in the different attack types. For each type, the p-value was calculated. The p-value was equal to zero for the CP, RP, ROP, ES, and BCM classifications, whereas for the COP attack, it was 9.3e-183. Based on these results, it was evident that there is a significant statistical difference between the two models, as the p-value was equal to or near zero in all types. This comparison highlights the substantial change in model behavior provided by the inclusion of the RSSIConf feature.

**Table 15:** The contingency table of the RF-FV2 and RF-FV2’ models in the different attack types.

			RF-FV2’	
			Correct	Misclassified
CP	RF-FV2	Correct	327,651	66,374
		Misclassified	0	84,391
COP	RF-FV2	Correct	471,313	2,282
		Misclassified	735	4,086
RP	RF-FV2	Correct	475,614	2,529
		Misclassified	65	575
ROP	RF-FV2	Correct	437,292	14,957
		Misclassified	7,764	19,564
ES	RF-FV2	Correct	375,597	27,050
		Misclassified	6,986	70,020
BCM	RF-FV2	Correct	2,196,382	36,116
		Misclassified	23,788	138,559

### Deployment

The proposed model is designed for deployment in a distributed manner, where each vehicle is equipped with its own pre-trained classifier capable of evaluating received messages in real time without relying on a centralized decision-making entity. The RF-FV2 model is well-suited for such distributed VANET environments due to the computational efficiency of RF classifiers, which impose relatively low processing overhead during the inference phase^55^.

**Fig. 9:**
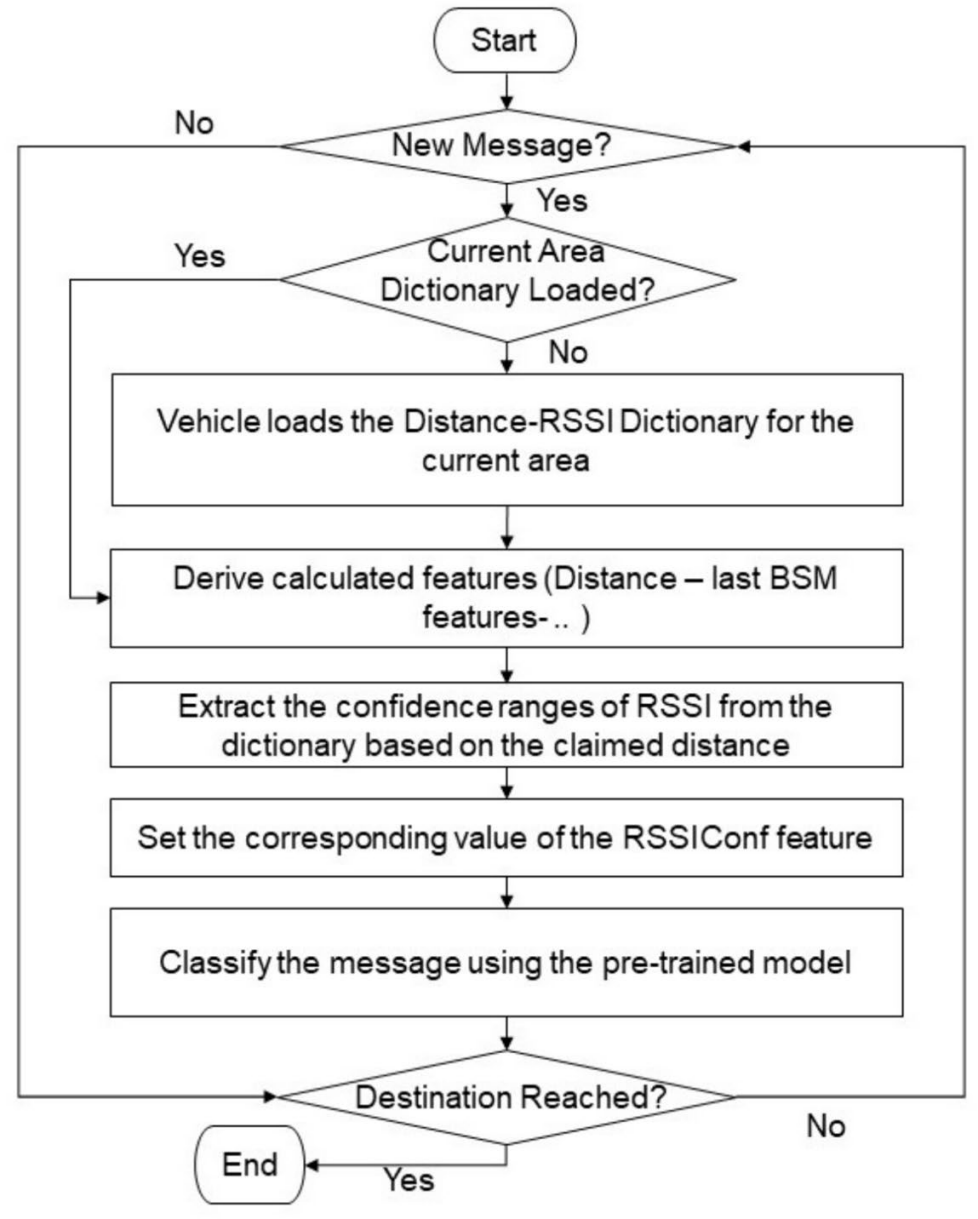
The operational workflow of the proposed model.

While earlier sections describe the proposed methodology and feature construction at a conceptual level, Fig. 9 provides an operational, deployment-oriented view of the real-time message processing workflow executed within each vehicle. Upon receiving a new message, a vehicle first verifies whether the Distance-to-RSSI dictionary corresponding to its current geographic area is already available. If not, the appropriate dictionary is loaded; this dictionary contains confidence intervals of RSSI values mapped to different distance ranges and confidence levels.

Subsequently, the required features are computed. To support these computations, each vehicle maintains a buffer storing the most recent message received from each sender. This buffer is essential for extracting time-dependent features, such as variations in the sender’s reported position and speed over successive messages. Based on the distance claimed in the received message, the pre-loaded dictionary is queried to obtain the corresponding RSSI confidence ranges at three different confidence levels. The observed RSSI value is then compared against these ranges to compute the RSSIConf feature. Finally, the full feature vector, including RSSIConf, is fed into the pre-trained classification model. Subsequently, the model outputs a decision indicating whether the received message is legitimate or falsified.

## Conclusion and future work

This paper presents a distributed ML-based detection scheme for position falsification attack detection. The proposed scheme implements a new feature called RSSIConf. The RSSIConf feature depends on calculating the RSSI confidence intervals at 90%, 95%, and 99% confidence levels for various distance ranges. Then, the RSSI feature of each received BSM is compared to the calculated RSSI confidence intervals at the three confidence levels for the claimed sender-receiver distance range, and a value is assigned to the RSSIConf feature based on this comparison.

The proposed model was evaluated by training and testing four ML algorithms—RF, KNN, XGB, and MLP—using three feature vectors (FV1, FV2, and FV3), which included the RSSIConf feature and a set of other selected and derived features. Then, the classification performance of all implemented models was evaluated, and the results show that the model implemented using FV2 features to train the RF classifier (RF-FV2) obtained the best performance among other developed models.

For the CP attack, the RF-FV2 model achieved near-perfect classification performance. In the case of COP and RP attacks, the model attained 99.9% accuracy and F1-score. For ROP attack detection, RF-FV2 demonstrated strong performance, achieving 99.7% accuracy and a 99.64% F1-score. Similarly, the model maintained high detection capability for the ES attack, with an accuracy of 99.89% and an F1-score of 99.87%. Finally, in the combined BCM classification scenario, RF-FV2 achieved 99.86% accuracy and a 99.84% F1-score.

Assessing the efficiency of the RF-FV2 model to existing models in the literature for five different types of position falsification attacks reveals that, under the evaluated simulation conditions, the model achieves an increase in accuracy that ranges between 2.66% and 13.26% and an enhancement in F1-score between 6.39% and 12.71%. Moreover, the model enhanced the binary classification of the combined five attacks by 0.76% and 0.74% in accuracy and F1-score.

To evaluate the contribution of the RSSIConf feature, a modified version of the original RF-FV2 model called RF-FV2’ was created. The modified model was trained using the same dataset, algorithm, and feature set as the original RF-FV2, excluding the RSSIConf feature. Both models were subsequently evaluated against the same dataset. The findings showed that the RSSIConf feature significantly improved detection accuracy, particularly for the CP attack, by 20.16%. Furthermore, attacks such as ES and ROP demonstrated accuracy increases of 5.23% and 1.63%, respectively. The F1-score also showed significant improvement, increasing by 83.89% for the CP attack, 10.49% for the ES attack, and 2.16% for the ROP attack.

While the proposed model demonstrates promising results, several limitations remain that warrant further investigation. The reported performance is based on simulation-generated data under controlled conditions, and although the VeReMi dataset provides multiple independent repetitions, it remains a homogeneous benchmark that may not fully capture the variability of real-world VANET environments. In addition, the practical deployment of the RSSIConf feature relies on the availability and maintenance of a precomputed Distance-to-RSSI dictionary, the efficient distribution and updating of which remains a challenge. Future work may focus on validating the proposed model using alternative simulation frameworks and real-world vehicular traces, as well as investigating efficient dictionary dissemination strategies. Moreover, to enhance the robustness and generalizability of the approach, future research could investigate its effectiveness against a broader range of attacks and explore ensemble or hybrid learning techniques.

## Data Availability

The dataset used in this study is publicly available at https://github.com/VeReMi-dataset/VeReMi.
